# Home-Based Individualized Alpha Transcranial Alternating Current Stimulation Improves Symptoms of Obsessive-Compulsive Disorder: Preliminary Evidence from a Randomized, Sham-Controlled Clinical Trial

**DOI:** 10.1155/2023/9958884

**Published:** 2023-08-31

**Authors:** M. Prabhavi N. Perera, Neil W. Bailey, Oscar W. Murphy, Sudaraka Mallawaarachchi, Caley Sullivan, Aron T. Hill, Paul B. Fitzgerald

**Affiliations:** ^1^Central Clinical School, Monash University, Wellington Road, Clayton, VIC 3800, Australia; ^2^School of Medicine and Psychology, Australian National University, Canberra, ACT 2600, Australia; ^3^Monarch Research Institute Monarch Mental Health Group, Sydney, NSW, Australia; ^4^Bionics Institute, East Melbourne, VIC 3002, Australia; ^5^Department of Biostatistics, Faculty of Medicine, University of Oslo, 0372 Oslo, Norway; ^6^Cognitive Neuroscience Unit, School of Psychology, Deakin University, Geelong, VIC 3220, Australia

## Abstract

Obsessive-compulsive disorder (OCD) is a debilitating mental health condition that is largely resistant to conventional treatments, such as pharmacotherapy and behavioural interventions. Individualized noninvasive brain stimulation techniques such as transcranial alternating current stimulation (tACS) might be capable of successfully treating OCD through modulation of dysfunctional neural circuitry. A randomized, double-blind, sham-controlled, pilot clinical trial involving 25 OCD patients was conducted to investigate the efficacy of tACS in improving OCD severity. Treatments targeting the medial prefrontal cortex (mPFC) were self-administered at home for 6 weeks with a 3-month follow-up. Within the active group, each treatment was delivered at an individualized peak alpha frequency for 30 minutes, while the sham group received 2 blocks of 2-minute treatments at 25 Hz. The clinical severity of OCD and potential symptom improvements were quantified using serial measurements of the Yale-Brown Obsessive-Compulsive Scale (YBOCS), and a linear mixed model analysis was performed to estimate the time-condition effect. There was a significant time-condition interaction in the YBOCS from baseline to 6 weeks (*p* < 0.0001), indicating that active alpha-tACS was significantly superior to sham in improving OCD severity. A trend-level effect remained at the 3-month follow-up, suggestive of a sustained level of improvement. Additionally, depressive symptoms also showed a significant improvement from baseline to follow-up. Our findings suggest that a six-week, home-based treatment course of individualized alpha-tACS targeting the mPFC is capable of improving OCD symptoms. Further large-scale clinical trials are required to definitively establish tACS as a therapy for OCD.

## 1. Introduction

Obsessive-compulsive disorder (OCD) is a severely debilitating mental health condition with a lifetime prevalence of 2-3% [1, 2]. It is reported that individuals with OCD have a significantly reduced quality of life with an increased likelihood of suicidality [3, 4]. OCD is characterized by repetitive, unwanted, intrusive thoughts (obsessions), which often lead to repetitive behaviours and rigidly applied rituals or mental acts (compulsions). If left untreated, OCD typically presents as a chronic disorder, which follows a waxing and waning course over time [5]. The first-line treatments for OCD include pharmacological therapy with selective serotonin reuptake inhibitors (SSRI) and cognitive behavioural therapy (CBT). However, poor response to these therapeutic modalities often leads to nonadherence [6]. Therefore, it is vital to investigate and trial novel treatments based on the limited existing knowledge on the pathophysiology of OCD.

Noninvasive brain stimulation (NIBS) techniques have attracted increased interest in recent years as new avenues for the treatment of OCD. Repetitive transcranial magnetic stimulation (rTMS) is a NIBS technique that utilizes a magnetic field to create a powerful and rapidly changing targeted electrical current in cortical tissue, causing neurons within the stimulated area to depolarize [7]. A recent meta-analysis reported that the therapeutic effects of rTMS are superior to sham stimulation in OCD [8]. However, rTMS has the rare potential to cause seizures as a side effect [9], and rTMS administration requires large medical equipment, which can only be accommodated within clinical environments. This is a major drawback in OCD groups, as their disease-related fears might prevent them from regular attendance.

Transcranial alternating current stimulation (tACS) is another NIBS technique that applies oscillatory electrical currents to the brain to influence cortical excitability [10]. The devices used to deliver tACS are typically small and portable, thus facilitating home-based treatment, a factor that is pivotal for treating OCD groups. The mode of action of tACS is thought to be via modulation of brain oscillations through neuronal entrainment in a frequency-specific manner [11]. Entrainment refers to the temporary alignment of intrinsic oscillatory brain activity to external electrical, magnetic, or sensory stimulation [12]. Previous research has suggested that entrainment is more effective when tACS is administered at an individualized frequency (i.e., personalized to each individual's endogenous neural oscillations) within the frequency band of interest [13, 14]. Moreover, tACS is known to induce plastic changes to the brain in the form of spike-timing-dependent plasticity (STDP) [13]. In STDP, brain circuits that resonate at a frequency similar to the repetitive external stimulation are selectively strengthened, such that once the stimulation is ceased, changes can persist resulting in enduring changes in neural activity at the resonance frequency of these circuits [15]. The reported aftereffects of alpha-tACS have been detected 70 minutes poststimulation [16]. tACS is reported to have a good safety and tolerability profile with relatively minor side effects, such as skin irritation under the electrode pads, headaches, and phosphene perception [17].

Electroencephalographic (EEG) studies have shown that OCD is associated with an array of electrophysiological differences in brain activity when compared to healthy controls (HC) [18]. Among the most consistently reported differences were that OCD groups have increased frontal and temporal oscillatory power in the delta and theta frequency bands, as well as decreased alpha oscillatory power [19–21]. It is known that theta and alpha oscillations play a crucial role in maintaining functional connectivity in the frontal-striatal-thalamic (FST) circuit [22]. As such, these findings might reflect pathophysiological changes in the FST circuitry, leading to poor functional connectivity between these brain regions [23, 24], which might underpin the clinical symptomatology of OCD. Thus, therapeutic interventions, such as tACS, that can modulate oscillatory activity at specific frequencies known to be disrupted in OCD might be capable of improving clinical symptoms.

The first study that explored tACS in OCD was a case series with seven participants, where gamma-tACS at 40 Hz was used to target the dorsolateral prefrontal cortex (DLPFC). Results showed a mean symptom reduction of 52% (28-86%) following treatment [25]. A recent case report also found an improvement in OCD symptoms as well as comorbid depressive symptoms in a female OCD patient following 10 sessions of gamma-tACS at 40 Hz to prefrontal regions [26]. A subsequent study that investigated the use of personalized beta-gamma tACS targeting the bilateral orbitofrontal cortex (OFC) in a large nonclinical sample (*n* = 128) of individuals with obsessive-compulsive behaviours reported significant attenuation of these behaviours after 5 treatment sessions [27].

Given this background, the main aim of the current study was to investigate the efficacy of individualized alpha-tACS in the treatment of OCD. Due to the scarcity of literature on the optimal tACS treatment parameters to use, we determined the target brain region and stimulation frequency based on pathophysiological evidence. The medial prefrontal cortex (mPFC) was chosen as the target brain region as it is known to be a vital element of the FST circuit. It is known that the mPFC is hyperactive in OCD, which is most evident from EEG activity within slow frequencies, such as the delta and theta bands [28]. Therefore, modulating this excessive slow wave activity by externally applying faster alpha oscillations at the individualized peak alpha frequency (IAF) might improve the underlying oscillatory anomalies. The primary hypothesis was that individuals with OCD would show a significantly greater improvement in clinical severity with individualized alpha-tACS when compared to sham stimulation. This is the first randomized, double-blind, sham-controlled clinical trial investigating the therapeutic use of tACS in a clinical OCD sample. Furthermore, it is noteworthy that this is the first instance of using individualized alpha-tACS with home-based treatments in an OCD sample.

## 2. Methods

### 2.1. Participants

A total of 25 male and female participants aged between 18 and 65 years were recruited through doctor referrals and Internet/poster advertisements from the state of Victoria, Australia. The required sample size calculation is presented in Supplementary Material S1. Recruitment commenced in September 2020, and data collection was completed in August 2022. The clinical trial received ethics approval from the Monash Health Human Research Ethics Committee and was registered on the Australian New Zealand Clinical Trials Registry (trial ID: ACTRN12620000748910). Verbal and written explanation of the nature of all procedures was provided to the participants prior to obtaining informed, written consent. Participants were reimbursed for time and travel expenses related to their participation. The clinical trial was conducted in accordance with the latest version of the Guidelines for Good Clinical Practice [29].

Individuals with a diagnosis of OCD according to the International Classification of Diseases-10^th^ revision [30] or DSM-IV/V were included [5]. Exclusion criteria included scoring <17 on the baseline Yale-Brown Obsessive-Compulsive Scale (YBOCS), being diagnosed with another mental health condition other than depression and anxiety, the presence of an unstable medical/neurological disorder, being pregnant/breastfeeding, and the presence of metal implants anywhere in the head, except inside the mouth. Participants were eligible to participate regardless of their medication status but were required to be on a stable dose for at least 6 weeks prior to the study. They must also not have initiated or ceased medications in the 6 weeks preceding the study.

### 2.2. Study Design and Randomization

**Figure 1:**
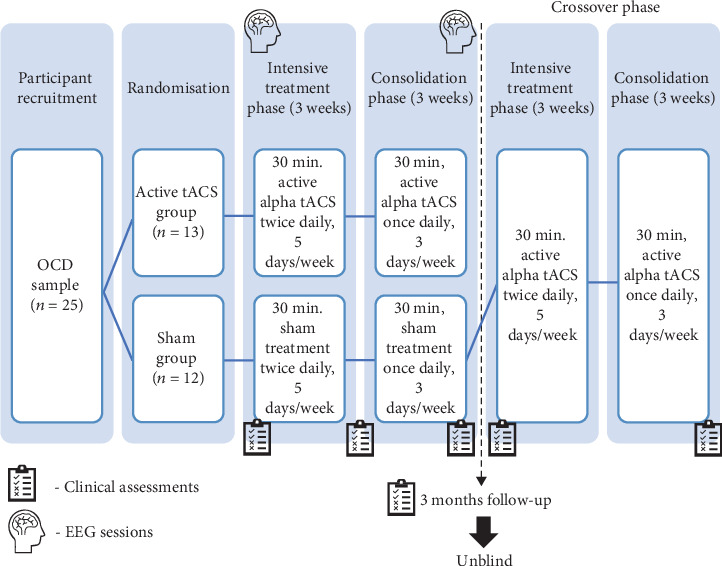
Study design of the clinical trial. Note: initially, the included OCD sample was randomly allocated to the active and sham groups. All participants underwent tACS treatments for 6 weeks and were followed up for 3 months. All participants and investigators were unblinded at the end of the 3-month follow-up assessment. The sham group was offered an open-label crossover phase with a similar treatment regimen. OCD: obsessive-compulsive disorder; tACS: transcranial alternating current stimulation; EEG: electroencephalography.

This study was a 6-week, randomized, double-blind, sham-controlled clinical trial with an open-label crossover phase. All participants were administered either tACS or sham treatment in two phases: (1) the intensive phase: 3 weeks of twice daily treatments on five days per week and (2) the consolidation phase: immediately following the intensive phase, 3 weeks of once daily treatments on 3 days per week (every second day from Monday to Friday). This protocol was designed to achieve maximal effects of tACS during the intensive phase, while reducing the relapse rates associated with abrupt cessation of treatments [31]. A follow-up assessment was conducted 3 months following the termination of treatments. Both the investigator and the participants were unblinded at the end of the 3-month follow-up assessment. Participants who received active treatments were not required to participate in the crossover phase. Participants who received sham treatments initially were given the opportunity to receive active tACS treatments with the same regimen. Randomization of participants into intervention first or sham first condition occurred via a computer-generated randomization list, and an investigator who had no direct contact with participants was responsible for programming the tACS devices prior to handing them over to participants. The participant, the clinical assessment conductor, and other investigators who had contact with participants were blind to the treatment condition.  Figure 1 summarizes the study design.

### 2.3. Clinical Instruments

Clinical assessments were conducted at baseline, 3 weeks (end of the intensive phase), 6 weeks (end of the consolidation phase), and a follow-up at 3 months posttreatment by a single investigator. Each assessment included a YBOCS [32], a Beck Anxiety Inventory (BAI) [33], and a Quick Inventory of Depressive Symptoms-Self Report (QIDS-SR) [34]. The Mini-International Neuropsychiatric Interview [35] was used at baseline to exclude major psychiatric comorbidities. Upon completing all tACS treatments, participants were requested to complete a questionnaire to assess the level of blinding success.

### 2.4. Electroencephalographic Recording and Preprocessing

Recordings of resting state and task-related EEG were performed at baseline, as well as 6 weeks postintervention. The baseline task-related EEG recordings were used to determine each participant's IAF required for the individualized tACS treatments. An analysis of the baseline and 6-week EEG data will be reported separately. EEG was recorded in a laboratory with constant levels of lighting and background noise from air conditioning. Participants were seated upright on a comfortable, padded chair and requested to stay awake and relaxed during recording. Additionally, participants were provided with an explanation of the EEG procedure, its safety, and instructions to minimize eye and muscle movements that may affect the recording.

Collection of EEG data was through an actiCHamp amplifier (Brain Products GmbH, Munich, Germany) using the BrainVision software (version 1.21.0303) and an EasyCap (Herrsching, Germany) with 64 Ag/AgCl electrodes, positioned in accordance with the international 10-20 system. CPz was used as the reference electrode and AFz was the ground. EEG data were recorded at a sampling rate of 1000 Hz, and impedances were kept below 5 k*Ω* throughout the session. No notch or online band-pass filtering was applied during recording.

### 2.5. Individualized Alpha Frequency Computation

Each participant's IAF was computed using EEG recordings obtained while participants performed a modified Sternberg working memory task [36]. This task was used as it has been shown that alpha power increases during its retention phase [37]. The steps involved in calculating the IAF are detailed in Supplementary Material S2, and the IAF of each participant is presented in Table S1. An IAF could not be detected for one participant, so in this instance, a standard 10 Hz stimulation frequency was used instead [38].

### 2.6. Tasks and Stimuli

For the modified Sternberg working memory task (Supplementary Figure S1), stimuli were presented using the Inquisit software [39] on a computer screen situated 75-85 cm from the participants' eyes. All participants were administered a short practice session with 5 trials before performing the task. In each trial, initially, a visually orienting cue was presented 1.8 s prior to presenting the letters comprising the memory set. Each letter list consisted of 8 consonants which were simultaneously displayed on the screen for 4 s. The termination of this display was followed by a delay period of 3 s, after which the probe letter was presented. Participants were asked to memorize the letter list and respond by pressing the left key if the probe was on the list and the right key if not. The subsequent trial began 1.8 s after the key press. One block of 27 trials was presented with a total duration of approximately 5 minutes.

### 2.7. Transcranial Alternating Current Stimulation

**Figure 2:**
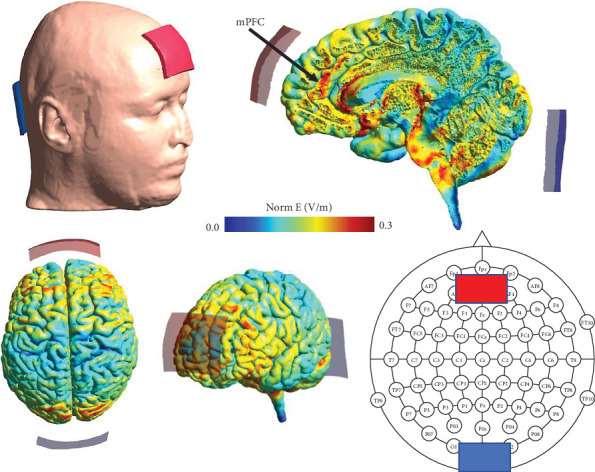
Electrode positioning and computational electric field modelling. Note: using computational electric field modelling, it was identified that the optimal electrode montage to target the bilateral medial prefrontal cortex (mPFC) was to position the tACS electrodes at AFz and Iz. Norm E: electric field intensity.

Administration of tACS was provided using custom-built BrightStim devices (Airtronic Circuits, Melbourne, Australia) that consisted of a handheld battery-driven unit that delivers a current-controlled voltage across two electrodes made of conductive rubber encased in commercially supplied saline-soaked sponges. The electrodes were positioned on the scalp using clips attached to a fitted cap. Two rectangular rubber electrodes encased in 7 cm × 6 cm sponges (contact area 42 cm^2^) were positioned at the 10-20 EEG system locations of AFz and Iz. This montage was chosen based on computational electric field models simulated using the SimNIBS software (version 2.0.1) [40] using the *ernie* head model included with the software, which indicated sufficient delivery of current to the target brain region, mPFC (Figure 2).

In the active condition, treatment was administered at the IAF of each participant with a peak-to-peak intensity of 1.5 mA for 30 minutes, with a 10 s ramp-up and a 10 s ramp-down. In the sham condition, current was delivered at an intensity of 1.5 mA in the first and last 2 minutes of the 30-minute session (10 s ramp-up/ramp-down) at a frequency of 25 Hz to emulate sensations associated with tACS administration without producing any enduring physiological effects.

The first treatment of each participant was administered under direct supervision of an experienced investigator. After training on self-administration and once each participant demonstrated they could implement the stimulation independently, participants were permitted to administer treatments at home. Additional remote supervision and support were provided via video and telephone communication. Participants were asked to relax and stay awake during the treatment. Participants were allowed to watch light television or listen to a podcast; however, they were requested to avoid drinking coffee/tea for at least 2 hours prior to the treatment and to refrain from reading, using a computer or mobile phone, engaging in physical activity, sleeping, or meditating during treatments. The tACS devices were programmed to save a log of the delivered treatments, which could be accessed by the investigator once the devices are returned.

### 2.8. Statistical Analysis

All statistical analyses were conducted using R [41]. Descriptive statistics were calculated for demographic variables using robust independent sample *t*-tests [42] from the WRS2 package [43]. A likelihood ratio test based on linear mixed model analysis was performed using the lme4 package in R [44] to assess the YBOCS variation with time. Further details of this analysis are provided in Supplementary Material S3. Secondary analyses were conducted on the self-reported questionnaire scores of the BAI and QIDS-SR using a similar approach. These results were corrected for experiment-wise multiple comparisons using the Benjamini and Hochberg false discovery rate (FDR) method [45]. To enable comparisons with future research, we have reported both the original and FDR-corrected *p* values. Additionally, a linear regression analysis was performed between the YBOCS difference from baseline to 6 weeks in the active group and the age, duration of illness, and baseline YBOCS to identify any associations. The blinding success was assessed with the blinding index (BI) package on R [46]. Details of this analysis are provided in Supplementary Material S4.

## 3. Results

### 3.1. Demographic and Clinical Data

At the beginning of the trial, the active and sham groups comprised 13 and 12 participants, respectively. The baseline demographic and clinical characteristics did not differ between the two groups (Table 1). Several participants dropped out at varying stages of the study, resulting in a total of 17 participants (9 active and 8 sham) that completed all treatments. The reasons for dropouts included technical difficulties in operating the tACS device (4 participants), time constraints (2 participants), and personal reasons (2 participants). However, all 25 participants were included in the final mixed model analysis. Supplementary Figure S2 depicts the CONSORT flow diagram and summarizes the participant dropouts at different stages. The CONSORT checklist for randomized clinical trials is presented in Supplementary Table S4. Concerning the safety of tACS, no serious adverse events were observed during the study. A detailed safety profile is included in Supplementary Table S5. Upon assessing the available treatment logs, all participants were found to be adherent to the treatment schedule.

### 3.2. Clinical Results

**Figure 3:**
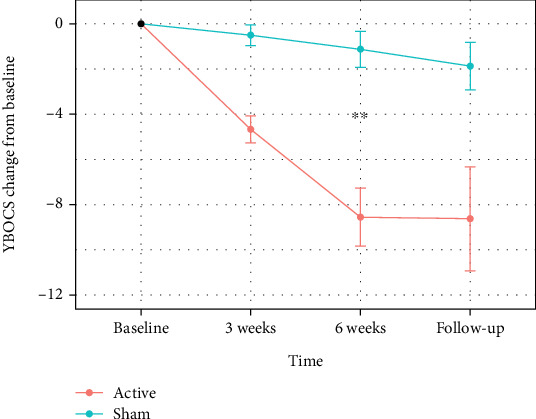
Difference in the Yale-Brown Obsessive-Compulsive Scale scores with time. Note: mean difference in YBOCS scores at each timepoint in the active and sham groups with standard error bars. The active group has shown a larger difference from baseline when compared to sham. Post hoc tests indicate that the difference in YBOCS means was significant at the 6-week timepoint (*p* = 0.009). YBOCS: Yale-Brown Obsessive-Compulsive Scale; ⁣^∗∗^*p* < 0.01.

The linear mixed model analysis of the YBOCS scores revealed a significant time-condition interaction from baseline to 6 weeks (*χ*^2^ = 32.49, *p* < 0.0001), indicating that the active treatment group showed a significantly greater reduction in YBOCS compared to sham. Additionally, the time-condition interaction was not significant from 6 weeks to follow-up (*χ*^2^ = 0.67, *p* = 0.412). Within post hoc tests, there was a significant difference between the active and sham conditions in the mean YBOCS at 6 weeks and at a trend level at 3 weeks and the 3-month follow-up (Table 2). Change in the YBOCS from baseline to the 3-month follow-up in each participant is shown in Supplementary Figure S3.  Figure 3 illustrates the reported improvement.

There was a significant group-by-time interaction in the QIDS-SR scores from baseline to the 3-month follow-up (*χ*^2^ = 4.42, *p* = 0.036), indicating that the active group showed a significantly greater reduction in QIDS-SR compared to the sham. This interaction was not significant for the BAI scores (*χ*^2^ = 0.02, *p* = 0.887). Time-condition interactions for QIDS-SR and BAI for all timepoint differences are depicted in Supplementary Table S2. Following FDR correction of the baseline to follow-up data, the time-condition interaction for YBOCS remained significant (*p* = 0.009), while the time-condition interactions within the QIDS-SR (*p* = 0.054) and BAI (*p* = 0.887) were not significant.

The participants were found to be sufficiently blinded with a James BI estimate of 0.64 (confidence interval (CI): 0.42 to 0.86) and Bang BI estimates for active: 0.11 (CI: -0.37 to 0.59) and sham: -0.13 (CI: -0.78 to 0.52). The regression analysis did not find a significant correlation between the YBOCS improvement and age, duration of illness, or baseline YBOCS in the active group (Supplementary Material S5 and Figure S4). The results of the open-label crossover phase have been presented in Supplementary Material S6 and Table S3.

## 4. Discussion

The present study reported findings from the first home-based randomized, double-blind, sham-controlled pilot clinical trial investigating the therapeutic use of individualized alpha-tACS in OCD. We found that alpha-tACS targeting the mPFC, delivered over 6 weeks, reduced clinical severity of OCD compared to sham stimulation. There was also a trend-level effect suggesting that this improvement was sustained at the 3-month follow-up point. Our findings also suggest that there may be an improvement of comorbid depressive symptoms with tACS, although this was not significant after correction for multiple comparisons. We did not find any correlation between the YBOCS improvement and age, duration of illness, or baseline YBOCS score.

Our findings align with three previous studies that used tACS in the treatment of OCD [25–27], all of which reported a significant improvement in OCD symptoms following tACS therapy. However, Klimke et al. [25] conducted their study in the form of a case series and therefore did not include a control group. Similarly, the study by Haller et al. [26] was a case report with findings from one individual with OCD. Although Grover et al. [27] recruited a large sample (*n* = 128), this consisted of nonclinical participants who were assessed based on obsessive-compulsive behaviours. Our study was the first randomized, double-blind, sham-controlled clinical trial to investigate the efficacy of tACS in OCD.

Our study was also the first to use individualized alpha-tACS in an OCD sample. tACS is thought to operate by neuronal entrainment in a frequency-specific manner [11], which is more effective when the external stimulation occurs at an individualized frequency [13, 14]. Therefore, the clinical improvement seen in our study may be due to maximally enhancing the alpha band activity. This may have resulted in an overpowering of the excessive slow wave activity that is implicated in the pathophysiology of OCD [18]. Furthermore, the improvement in OCD symptom severity was found to be sustained at the 3-month follow-up assessment, which indicates that treatment effects are likely to have been enduring in nature. This may be due to tACS-induced STDP [13], leading to enhanced neural activity within the selectively strengthened circuits resonating at the IAF.

Our study targeted the mPFC using individualized alpha-tACS treatments. These parameters were chosen based on the FST circuitry dysfunction theory of OCD [22]. It is known that a dysfunctional FST network leads to poor functional connectivity between the brain regions involved in this network [47]. The mPFC, being a vital part of the FST circuit, is thought to be hyperactive as a result of this dysfunction, leading to excessive oscillatory activity in the delta and theta frequency bands [28]. Therefore, modulating this excessive slow wave activity in the mPFC by externally applying a higher frequency (i.e., alpha) may resolve the dysfunctional neural activity. Our preliminary findings suggest significant improvement in OCD symptom severity, which provides motivation to further investigate mPFC as a potential target in future research.

Our findings suggest that comorbid depressive symptoms may also have improved with tACS treatments. There is evidence showing a linkage between the pathophysiology of depressive symptoms in major depressive disorder (MDD) and comorbid depression in OCD, with similar functional alterations seen in the DLPFC region in both conditions [48]. A recent randomized, double-blind clinical trial reported that, compared to sham stimulation, alpha-tACS at 10 Hz delivered to the DLPFC significantly improved clinical symptoms in an MDD sample [49]. As the DLPFC is an adjacent brain structure to the mPFC, it is possible that the tACS treatments administered in our study may have also stimulated the DLPFC leading to the improvement of depressive symptoms in our OCD sample. Alternatively, it may be that improvement in the symptoms of OCD led to an improvement in depression symptoms as the quality of life was improved.

Furthermore, there are two notable findings on the trend of the comorbid depressive symptoms in our OCD sample. Firstly, the baseline QIDS-SR scores of the active group were higher than the sham group (although at a nonsignificant level). Secondly, the QIDS-SR scores varied only slightly throughout the tACS treatment period and improved considerably at the 3-month follow-up point. It is known that individuals with MDD only experience relief from regular anti-depressant therapies 4-6 weeks after commencement, which is likely to be explained by the gradual regulatory effects of these therapies on neural plasticity [50]. Therefore, a similar mechanism may have caused the depressive symptoms to be relieved slower than OCD symptoms in our sample. However, caution is warranted regarding this conclusion, and further research is required to provide a more rigorous test of the potential for tACS to reduce depression in OCD.

This was also the first study to use home-based tACS treatments in an OCD sample, which is particularly beneficial in OCD as regular attendance to clinical environments might be symptom-provoking, leading to nonadherence. Furthermore, it has been identified that other forms of therapies for OCD such as CBT may be more effective when performed at the patient's home [51]. Additionally, a feasibility and acceptability study of transcranial direct current stimulation (a different NIBS technique) in OCD reported that home-based treatments may be more effective in the treatment of OCD [52]. Our study appears to corroborate these findings, as a majority of the participants completed the trial and informally provided the researcher with positive feedback about their experience.

### 4.1. Limitations and Future Directions

The present study included a small sample size of 25 participants at baseline, and a considerable number of dropouts occurred at various stages of the study. Nevertheless, despite the reduction in power this may have caused, the clinical efficacy we observed is encouraging to initiate further investigations of a similar nature in larger samples. We included participants with a diagnosis of OCD based on either ICD-10 or DSM IV/V criteria. Although these criteria are generally comparable, there are subtle differences, which may have caused heterogeneity in the study population.

While our findings showed a significant improvement of OCD symptoms from baseline to 6 weeks, only a trend effect was present at the follow-up timepoint compared to the improved scores at the 6-week end of the treatment timepoint. Additionally, there was one further dropout during the follow-up period resulting in an even smaller sample size at 3 months. As such, future research with larger sample sizes is justified to further explore the persistence of the treatment effect. We used individualized alpha-tACS in this study based on the previous evidence of altered oscillatory activity in OCD [19]. Investigating whether this altered activity was restored posttreatment may be helpful in interpreting the mechanism of action of tACS in OCD. Furthermore, it is known that several event-related potentials (ERP), such as the error-related negativity and N200, are altered in OCD when compared to HC [18, 53]. There is some evidence that these ERPs are generated at least partly by oscillations in the theta frequency band [54]. Future studies could also incorporate tACS in other frequency bands and incorporate ERP findings from baseline to posttreatment to explore the underlying pathophysiology of oscillatory and ERP activity in OCD.

### 4.2. Conclusions

OCD is a mental health condition leading to significant distress and poor quality of life. Individuals with OCD are largely nonresponsive to the current first-line treatments. Therefore, noninvasive brain stimulation methods such as tACS are increasingly being trialed for OCD in an attempt to directly address the neural activity that differs between OCD and healthy individuals. The present study conducted a home-based randomized, double-blind, sham-controlled pilot clinical trial with 25 individuals with OCD to investigate the efficacy of individualized alpha-tACS targeting the mPFC. We found a significant improvement in the OCD symptom severity from baseline to 6 weeks in the active tACS condition, when compared to sham. There was also a trend-level effect suggesting an improvement in depressive symptoms with tACS treatments. In conclusion, this pilot study reports that individualized alpha-tACS targeting the mPFC is superior to sham stimulation in reducing OCD symptoms. Our findings provide promising initial evidence for the potential therapeutic efficacy of home-based, individualized alpha-tACS in treating OCD and justify further clinical trials utilizing larger samples of participants.

## Figures and Tables

**Table 1:** Baseline demographic and clinical characteristics of participants.

Variable	Active (*n* = 13)			Sham (*n* = 12)			Test statistic (*p* value)
	Mean	SD	*n*	Mean	SD	*n*	
Gender (M/F)			7/6			5/7	*χ* ^2^ = 0.36, *p* = 0.55
Age (years)	36.31	14.43	13	36.17	12.04	12	*t* = 0.03, *p* = 0.98
Age at onset (years)	23.08	11.91	13	26.33	7.3	12	*t* = 0.82, *p* = 0.42
Duration of illness (years)	13.23	11.88	13	9.83	7.55	12	*t* = 0.85, *p* = 0.41
YBOCS (total)	28.62	4.57	13	27.33	2.84	12	*t* = 0.83, *p* = 0.41
YBOCS-obsessions	14.23	2.31	13	13.50	1.00	12	*t* = 1.01, *p* = 0.32
YBOCS-compulsions	14.38	2.57	13	13.83	2.25	12	*t* = 0.57, *p* = 0.57
BAI	18.69	10.79	13	15.09	5.96	11^†^	*t* = 0.99, *p* = 0.34
QIDS-SR	11.46	5.49	13	8.64	3.91	11^†^	*t* = 1.43, *p* = 0.17

OCD: obsessive-compulsive disorder; HC: healthy control; SD: standard deviation; M: male; F: female; R: right; L: left; S: single; M: married; YBOCS: Yale-Brown Obsessive-Compulsive Scale; BAI: Beck Anxiety Inventory; QIDS-SR: Quick Inventory of Depressive Symptoms-Self Report. ^†^BAI and QIDS-SR scores for one participant were unavailable due to a data collection issue.

**(a):** 

Mean scores of outcome measures and between-group comparisons					
Timepoint	Active		Sham		Test statistic (*p* value)
	Mean ± SD	*n*	Mean ± SD	*n*	
YBOCS					
Baseline	28.6 ± 4.57	13	27.3 ± 2.84	12	*t* = 0.83, *p* = 0.406
3 weeks	24.2 ± 4.86	12	27.5 ± 2.98	8	*t* = 1.90, *p* = 0.073
6 weeks	19.8 ± 5.80	9	26.9 ± 3.72	8	*t* = 3.03, *p* = 0.009^∗∗^
3-month follow-up	19.4 ± 8.63	8	26.1 ± 4.42	8	*t* = 1.97, *p* = 0.076
QIDS-SR					
Baseline	11.5 ± 5.49	13	8.6 ± 3.91	11	*t* = 1.47, *p* = 0.157
3 weeks	11.3 ± 7.71	9	8.3 ± 3.73	7	*t* = 1.04, *p* = 0.319
6 weeks	11.3 ± 8.14	9	8.1 ± 6.23	7	*t* = 0.89, *p* = 0.390
3-month follow-up	5.14 ± 2.67	7	8.9 ± 3.98	7	*t* = −2.05, *p* = 0.067
BAI					
Baseline	18.7 ± 10.80	13	15.1 ± 5.96	11	*t* = 1.03, *p* = 0.315
3 weeks	15.3 ± 14.70	10	18.0 ± 9.40	7	*t* = −0.46, *p* = 0.650
6 weeks	9.1 ± 4.94	8	18.0 ± 10.30	7	*t* = −2.08, *p* = 0.070
3-month follow-up	9.7 ± 3.68	7	15.6 ± 8.98	7	*t* = −1.60, *p* = 0.149

**(b):** 

YBOCS linear mixed model analysis results (group-by-time interaction)		
Duration	Chi-square value	*p* value
Baseline to 3 weeks	18.86	<0.0001⁣^∗∗∗^
Baseline to 6 weeks	32.49	<0.0001⁣^∗∗∗^
Baseline to 3-month follow-up	8.81	0.003⁣^∗∗^
3 weeks to 6 weeks	7.99	0.005⁣^∗∗^
3 weeks to 3-month follow-up	0.78	0.377
6 weeks to 3-month follow-up	0.67	0.412

SD: standard deviation; YBOCS: Yale-Brown Obsessive-Compulsive Scale; QIDS-SR: Quick Inventory of Depressive Symptoms-Self Report; BAI: Beck Anxiety Inventory. ⁣^∗^*p* < 0.05; ⁣^∗∗^*p* < 0.01; ⁣^∗∗∗^*p* < 0.001.

## Data Availability

The data used to support the findings of this study are restricted by the Monash Health Human Research Ethics Committee in order to protect patient privacy. Deidentified data are available from the corresponding author upon request.

## Supplementary Materials

Supplementary Materials Supplementary section S1 shows the sample size calculation. S2 describes the method used to calculate the IAF. Table S1 shows the IAF of each participant. Figure S1 presents the Sternberg working memory task design. Section S3 contains an explanation of the linear mixed model analysis. Figure S2 shows the CONSORT flow diagram of included participants and dropouts. Section S4 summarizes the method used to assess blinding success. Table S2 shows the group-by-time interaction of the linear mixed model analysis for QIDS-SR and BAI scores. Figure S3 presents the change in YBOCS from baseline to the 3-month follow-up in each participant. Section S5 and Figure S4 show the regression analysis between YBOCS difference and other parameters. Section S6 and Table S3 highlight the results of the open-label crossover phase. Table S4 contains the CONSORT checklist for randomized clinical trials. Table S5 presents the detailed safety profile of tACS.
