# Supplementary material for: Home-Based Individualized Alpha Transcranial Alternating Current Stimulation Improves Symptoms of Obsessive-Compulsive Disorder: Preliminary Evidence from a Randomized, Sham-Controlled Clinical Trial
Source: Depress Anxiety. 2023 Aug 31;2023:9958884. doi: 10.1155/2023/9958884 (PMC11921853; doi:10.1155/2023/9958884)
Supplement: Supplementary Materials — Supplementary section S1 shows the sample size calculation. S2 describes the method used to calculate the IAF. Table S1 shows the IAF of each participant. Figure S1 presents the Sternberg working memory task design. Section S3 contains an explanation of the linear mixed model analysis. Figure S2 shows the CONSORT flow diagram of included participants and dropouts. Section S4 summarizes the method used to assess blinding success. Table S2 shows the group-by-time interaction of the linear mixed model analysis for QIDS-SR and BAI scores. Figure S3 presents the change in YBOCS from baseline to the 3-month follow-up in each participant. Section S5 and Figure S4 show the regression analysis between YBOCS difference and other parameters. Section S6 and Table S3 highlight the results of the open-label crossover phase. Table S4 contains the CONSORT checklist for randomized clinical trials. Table S5 presents the detailed safety profile of tACS. [file 9958884.f1.pdf]

Supplementary Material

S1. Sample Size Calculation

Sample size (n_0) was calculated using the Cochran's formula [1]:

$$n_0 = \frac{Z^2 pq}{e^2},$$

where $e = 5.5\%$ denotes the margin of error, $p = 2\%$ is the estimated proportion of the population [2], $q = (1 - p)$ and $Z = Z_{\alpha/2}$ is the standard normal probability of $\alpha = 0.95$ confidence level, which yields the required sample size of 25.

S2. Individualised Peak Alpha Frequency Calculation

Peak individualised alpha frequency (IAF) was computed in MATLAB using a custom scripted pipeline. EEG recorded while performing the Sternberg working memory task was initially segmented into 8 second epochs [-3, 8] around the 'delay' marker (representing the beginning of the retention period). Data was pre-processed and cleaned in a fully automated fashion using the Harvard Automated Processing Pipeline for Electroencephalography (HAPPE) [3]. The overall processing time was optimised using the fastICA algorithm in place of the default extended-infomax independent component analysis (ICA) algorithm used in HAPPE [4], as previous research has demonstrated that these algorithms are effectively equivalent when used for removing artifacts [5].

Cleaned and epoched data was further reduced to 3 seconds in length, representing the period from stimulus presentation until the prompt to respond (after exclusion of the first and last 500 ms of the 4 s period of stimulus presentation). Epochs with incorrect responses were discarded, such that the dataset represented brain activity during memory retention periods that preceded only correct responses. The FCz

channel was used to estimate power in the alpha frequency band (8 – 12 Hz) for each epoch based on a power spectral density estimate using the pwelch method. Detection of a reliable alpha peak was ensured by automatically discarding 15% of trials that returned the lowest alpha power values.

An estimation of IAF was then calculated using the remaining high alpha power epochs, and a selection of frontocentral electrodes (F1, F3, F5, F7, Fz, F2, F4, F6, F8, FC1, FC3, FCz, FC2, FC4). This method of IAF detection employs the Savitzky-Golay filter approach described in detail by Corcoran et al. (2018) [6]. The IAF values of all participants are summarised in **Table S1.** If an IAF was not detected after two attempts at running the above algorithm, stimulation was given at a pre-determined frequency of 10 Hz [7].

Table S1 - Individualised alpha peak frequency of each participant

Participant ID	IAF Detected? (Yes/No)	IAF (Hz)
1	Yes	10.5
2	Yes	8.1
3	Yes	8.5
4	Yes	11
5	Yes	9.8
6	Yes	9.3
7	Yes	11.5
8	Yes	9.9
9	Yes	11.7
10	Yes	8.2
11	Yes	9.5
12	Yes	11.9
13	Yes	8.3
14	Yes	9.7
15	Yes	10.8
16	Yes	10.5
17	Yes	10.4

18	Yes	8.3
19	Yes	10
20	Yes	10.2
21	Yes	12
22	Yes	10.4
23	Yes	9
24	Yes	11.1
25	No	-
**Mean IAF (SD)**		**10.025 (1.202)**

Figure S1

Sternberg working memory task design

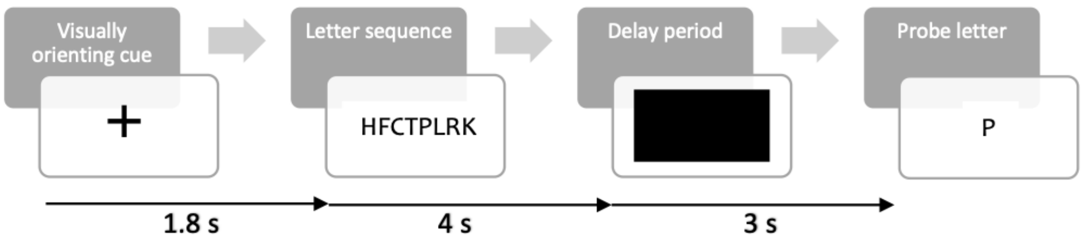

Note. Each trial begins with a visually orienting cue presented for 1.8s, followed by a letter sequence presented for 4s. After a delay period of 3s, the probe letter is presented. The participant is requested to memorise the letter sequence and respond whether the probe letter was in the sequence or not. The subsequent trial begins 1.8s after the key press.

S3. Linear Mixed Model Analysis

Likelihood ratio testing was used to specifically assess the time-condition interaction effect (i.e., measure the variation of YBOCS with treatment). In the null model, the outcome variable (YBOCS score) was modelled with two fixed effect predictors: (1) time (baseline, 3 weeks, 6 weeks, 3-month follow-up) and (2) condition (active, sham), and study participants were treated as a random effect. In the full model, an additional fixed effect predictor, time-condition interaction was used. A χ^2distributed statistic with

1-df was estimated from the log-likelihood ratio between the two linear mixed effect models.

Figure S2

CONSORT flow diagram of included participants and dropouts

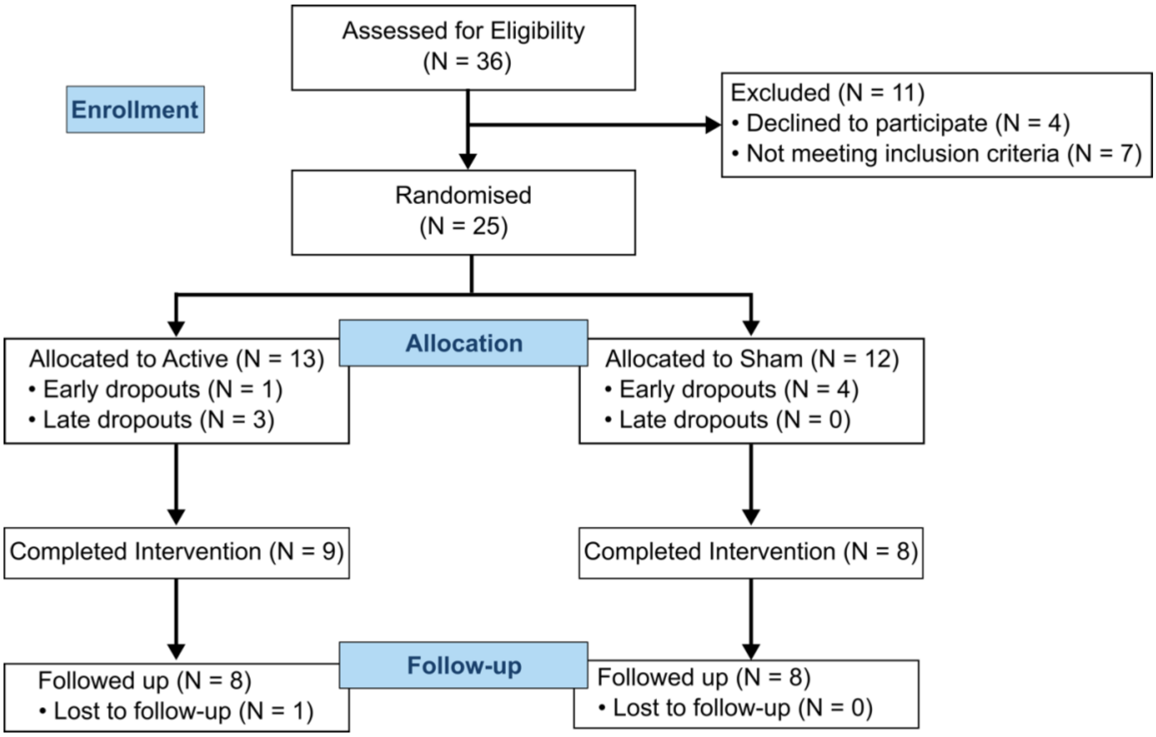

Note. CONSORT flow diagram illustrating the number of participants that were randomised, dropped out and the remaining participants at each phase of the study.

S4. Assessment of Blinding Success

The blinding success was assessed with the blinding index (BI) package on R [8], which computes 2 values: (1) James BI: provides a value for the overall blinding success, scaled to an interval of 0 to 1, 0 being complete lack of blinding and 1 being complete blinding [9]; (2) Bang BI: provides values for each treatment arm on a scale from -1 to 1, 1 being complete lack of blinding, 0 being adequate blinding and -1 being opposite guessing [10].

Table S2 – Group-by-time interaction of the linear mixed model analysis for QIDS-SR and BAI scores at each duration

Duration	Chi-square value	p-value
**QIDS-SR**		
Baseline to 3 weeks	0.68	0.409
Baseline to 6 weeks	1.51	0.220
Baseline to 3-month follow-up	4.42	0.036 *
3 weeks to 6 weeks	0.01	0.919
3 weeks to 3-month follow-up	7.96	0.005 **
6 weeks to 3-month follow-up	4.73	0.029 *
**BAI**		
Baseline to 3 weeks	0.11	0.735
Baseline to 6 weeks	0.32	0.571
Baseline to 3-month follow-up	0.02	0.887
3 weeks to 6 weeks	0.59	0.443
3 weeks to 3-month follow-up	0.04	0.851
6 weeks to 3-month follow-up	0.83	0.362

QIDS-SR – Quick Inventory of Depressive Symptoms-Self Report, BAI – Beck Anxiety Inventory
* $p < 0.05$, ** $p < 0.01$

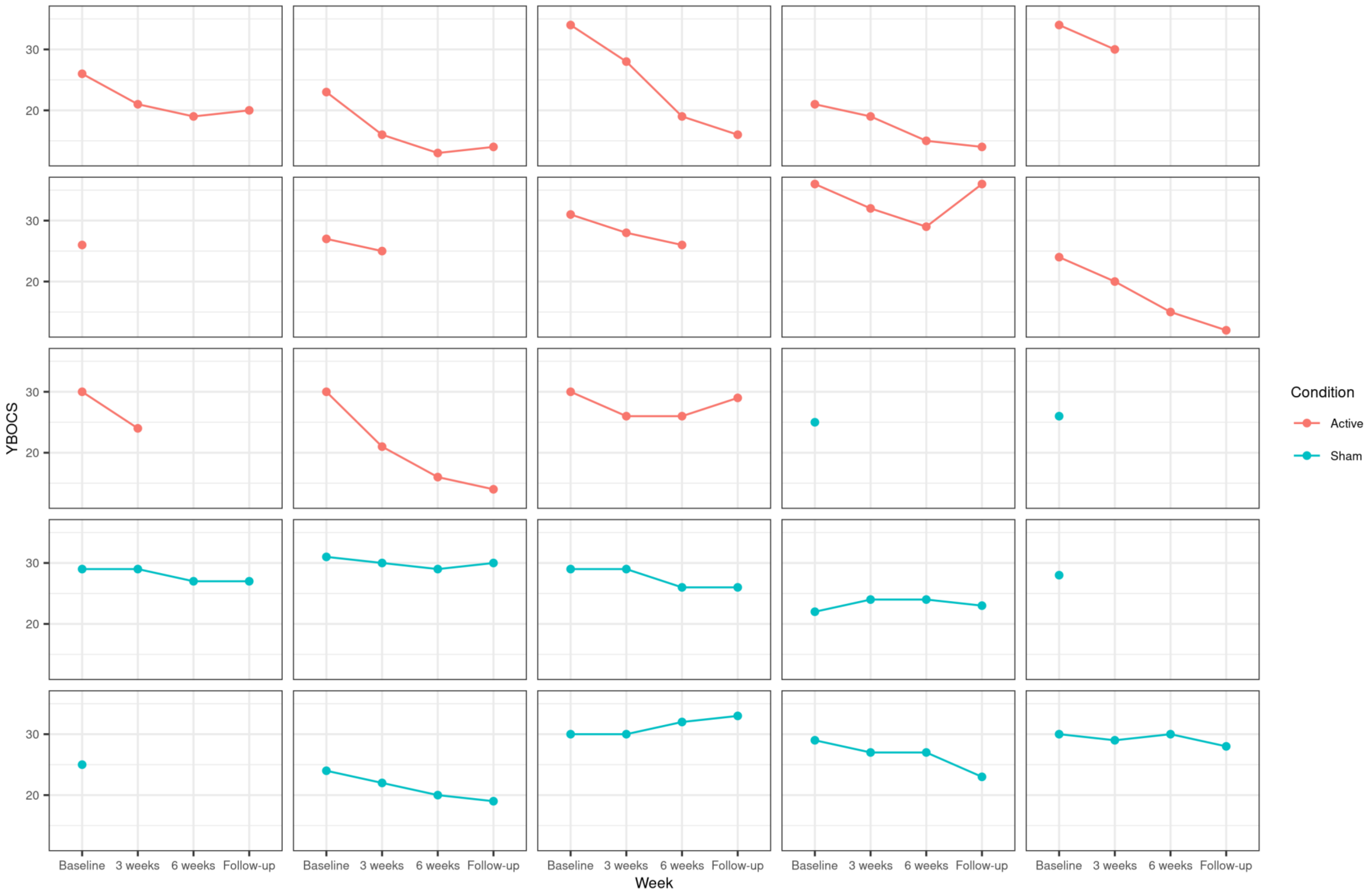

Figure S3 - Change in YBOCS from baseline to the 3-month follow-up in each participant

S5. Linear regression analysis

The regression analysis did not find a significant correlation between the YBOCS improvement and age, duration of illness or baseline YBOCS in the active group (Figure S4).

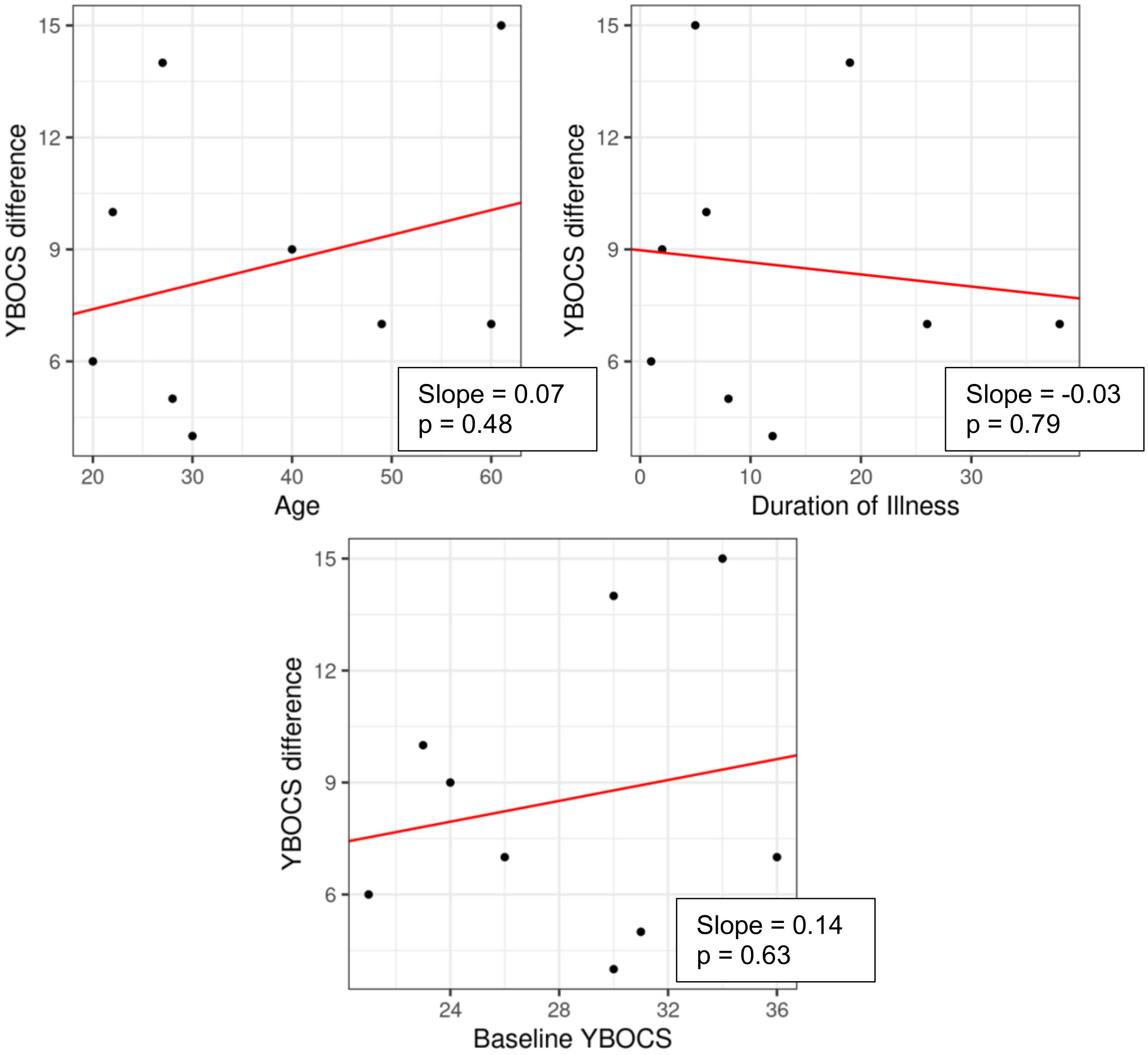

Figure S4 - Linear regression analysis between YBOCS difference in the participants of the active group and age, duration of illness and baseline YBOCS score.

S6. Open-label crossover phase results

Participants who received active treatments were not required to participate in the crossover phase. Participants who received sham treatments initially were given the opportunity to receive active tACS treatments with the same regime. Out of the 8 participants who completed treatments in the sham group, only 2 participants completed the crossover phase. Reasons for not taking up crossover treatments included time constraints and being an active participant of another clinical trial following the first phase. In the crossover phase, participants self-administered active tACS treatments at the individualised alpha frequency using the same regime used for the active group in phase 1. Assessments for clinical severity occurred at baseline and 6 weeks. The results of these assessments are summarised in Table S3.

Table S3 – Summary of clinical assessments in the crossover phase

	Baseline			6 weeks			Percentage change (%)		
	YBOCS	QIDS-SR	BAI	YBOCS	QIDS-SR	BAI	YBOCS	QIDS-SR	BAI
Participant 1	26	12	14	21	12	9	19.23	0	35.71
Participant 2	23	5	12	13	3	21	43.48	40	-75

YBOCS – Yale-Brown obsessive compulsive scale, QIDS-SR – Quick Inventory of Depressive Symptoms-Self Report, BAI – Beck Anxiety Inventory

Table S4 - CONSORT checklist for randomised clinical trials

CONSORT 2010 checklist of information to include when reporting a randomised trial*

Section/Topic	Item No	Checklist item	Reported on page No
**Title and abstract**			
	1a	Identification as a randomised trial in the title	1
	1b	Structured summary of trial design, methods, results, and conclusions (for specific guidance see CONSORT for abstracts)	2
**Introduction**			
Background and objectives	2a	Scientific background and explanation of rationale	3-4
	2b	Specific objectives or hypotheses	5
**Methods**			
Trial design	3a	Description of trial design (such as parallel, factorial) including allocation ratio	6
	3b	Important changes to methods after trial commencement (such as eligibility criteria), with reasons	-
Participants	4a	Eligibility criteria for participants	6
	4b	Settings and locations where the data were collected	5
Interventions	5	The interventions for each group with sufficient details to allow replication, including how and when they were actually administered	9-10
Outcomes	6a	Completely defined pre-specified primary and secondary outcome measures, including how and when they were assessed	7
	6b	Any changes to trial outcomes after the trial commenced, with reasons	-
Sample size	7a	How sample size was determined	-
	7b	When applicable, explanation of any interim analyses and stopping guidelines	-
Randomisation:			
Sequence generation	8a	Method used to generate the random allocation sequence	6
	8b	Type of randomisation; details of any restriction (such as blocking and block size)	6
Allocation concealment mechanism	9	Mechanism used to implement the random allocation sequence (such as sequentially numbered containers), describing any steps taken to conceal the sequence until interventions were assigned	6
Implementation	10	Who generated the random allocation sequence, who enrolled participants, and who assigned participants to interventions	6
Blinding	11a	If done, who was blinded after assignment to interventions (for example, participants, care providers, those	6

			6
		assessing outcomes) and how	6
	11b	If relevant, description of the similarity of interventions	9
Statistical methods	12a	Statistical methods used to compare groups for primary and secondary outcomes	10-11
	12b	Methods for additional analyses, such as subgroup analyses and adjusted analyses	10-11
**Results**			
Participant flow (a diagram is strongly recommended)	13a	For each group, the numbers of participants who were randomly assigned, received intended treatment, and were analysed for the primary outcome	11
	13b	For each group, losses and exclusions after randomisation, together with reasons	11
Recruitment	14a	Dates defining the periods of recruitment and follow-up	11
	14b	Why the trial ended or was stopped	-
Baseline data	15	A table showing baseline demographic and clinical characteristics for each group	12
Numbers analysed	16	For each group, number of participants (denominator) included in each analysis and whether the analysis was by original assigned groups	11
Outcomes and estimation	17a	For each primary and secondary outcome, results for each group, and the estimated effect size and its precision (such as 95% confidence interval)	12-14
	17b	For binary outcomes, presentation of both absolute and relative effect sizes is recommended	12-14
Ancillary analyses	18	Results of any other analyses performed, including subgroup analyses and adjusted analyses, distinguishing pre-specified from exploratory	14
Harms	19	All important harms or unintended effects in each group (for specific guidance see CONSORT for harms)	11
**Discussion**			
Limitations	20	Trial limitations, addressing sources of potential bias, imprecision, and, if relevant, multiplicity of analyses	17
Generalisability	21	Generalisability (external validity, applicability) of the trial findings	15-17
Interpretation	22	Interpretation consistent with results, balancing benefits and harms, and considering other relevant evidence	15-17
**Other information**			
Registration	23	Registration number and name of trial registry	5
Protocol	24	Where the full trial protocol can be accessed, if available	-
Funding	25	Sources of funding and other support (such as supply of drugs), role of funders	18

*We strongly recommend reading this statement in conjunction with the CONSORT 2010 Explanation and Elaboration for important clarifications on all the items. If relevant, we also recommend reading CONSORT extensions for cluster randomised trials, non-inferiority and equivalence trials, non-pharmacological treatments, herbal interventions, and pragmatic trials. Additional extensions are forthcoming: for those and for up to date references relevant to this checklist, see www.consort-statement.org.

Table S5 – Detailed Safety Profile of tACS

**Serious Adverse Events**		
None		
**Minor Adverse Events**		
**Type of Adverse Event**	**No. of participants experienced**	**Average Frequency of Adverse Event**
Headache	2 (8%)	14.1%
Phosphene perception	5 (20%)	44.62%
Tingling sensation beneath electrodes	4 (16%)	75.64%
Itching beneath electrodes	2 (8%)	51.28%

Note. Altogether, eight participants reported minor adverse events. The third column shows the average frequency of the adverse event of the participants who reported each minor adverse event.

REFERENCES

[1] J. Kotrlik and C. Higgins, "Organizational research: Determining appropriate sample size in survey research appropriate sample size in survey research," *Information technology, learning, and performance journal,* vol. 19, no. 1, p. 43, 2001.

[2] M. A. Fullana *et al.*, "Obsessions and compulsions in the community: prevalence, interference, help-seeking, developmental stability, and co-occurring psychiatric conditions," *American Journal of Psychiatry,* vol. 166, no. 3, pp. 329-336, 2009.

[3] L. J. Gabard-Durnam, A. S. Mendez Leal, C. L. Wilkinson, and A. R. Levin, "The Harvard Automated Processing Pipeline for Electroencephalography (HAPPE): standardized processing software for developmental and high-artifact data," *Frontiers in neuroscience,* vol. 12, p. 97, 2018.

[4] A. Hyvarinen, "Fast ICA for noisy data using Gaussian moments," in *1999 IEEE international symposium on circuits and systems (ISCAS)*, 1999, vol. 5: IEEE, pp. 57-61.

[5] N. Bailey *et al.*, "Introducing RELAX (the Reduction of Electroencephalographic Artifacts): A fully automated pre-processing pipeline for cleaning EEG data-Part 2: Application to Event-Related Potentials," *bioRxiv,* 2022.

[6] A. W. Corcoran, P. M. Alday, M. Schlesewsky, and I. Bornkessel-Schlesewsky, "Toward a reliable, automated method of individual alpha frequency (IAF) quantification," *Psychophysiology,* vol. 55, no. 7, p. e13064, 2018.

[7] M.-C. Davis *et al.*, "Medial prefrontal transcranial alternating current stimulation for apathy in Huntington’s disease," *medRxiv,* 2022.

[8] M. Schwartz, N. Mercaldo, and M. M. Schwartz, "Package ‘BI’," 2021.

[9] K. E. James, D. A. Bloch, K. K. Lee, H. C. Kraemer, and R. K. Fuller, "An index for assessing blindness in a multi-centre clinical trial: disulfiram for alcohol cessation—a VA cooperative study," *Statistics in medicine,* vol. 15, no. 13, pp. 1421-1434, 1996.

[10] H. Bang, L. Ni, and C. E. Davis, "Assessment of blinding in clinical trials," *Controlled clinical trials,* vol. 25, no. 2, pp. 143-156, 2004.
